# Auxetic Metamaterials for Biomedical Devices: Current Situation, Main Challenges, and Research Trends

**DOI:** 10.3390/ma15041439

**Published:** 2022-02-15

**Authors:** Vladislav A. Lvov, Fedor S. Senatov, Alnis A. Veveris, Vitalina A. Skrybykina, Andrés Díaz Lantada

**Affiliations:** 1 Center for Biomedical Engineering, National University of Science and Technology “MISIS”, Leninskiy pr. 4s1, 119049 Moscow, Russia; senatov@misis.ru (F.S.S.); mrechoru48@gmail.com (A.A.V.); vitalina.andreevna1211@gmail.com (V.A.S.); 2 Department of Mechanical Engineering, Universidad Politécnica de Madrid, José Gutiérrez Abascal 2, 28006 Madrid, Spain

**Keywords:** auxetics, metamaterials, biomedical devices, additive manufacturing, microfabrication, tissue engineering, computational modelling

## Abstract

Auxetic metamaterials are characterized by a negative Poisson ratio (NPR) and display an unexpected property of lateral expansion when stretched and densification when compressed. Auxetic properties can be achieved by designing special microstructures, hence their classification as metamaterials, and can be manufactured with varied raw materials and methods. Since work in this field began, auxetics have been considered for different biomedical applications, as some biological tissues have auxetic-like behaviour due to their lightweight structure and morphing properties, which makes auxetics ideal for interacting with the human body. This research study is developed with the aim of presenting an updated overview of auxetic metamaterials for biomedical devices. It stands out for providing a comprehensive view of medical applications for auxetics, including a focus on prosthetics, orthotics, ergonomic appliances, performance enhancement devices, in vitro medical devices for interacting with cells, and advanced medicinal clinical products, especially tissue engineering scaffolds with living cells. Innovative design and simulation approaches for the engineering of auxetic-based products are covered, and the relevant manufacturing technologies for prototyping and producing auxetics are analysed, taking into consideration those capable of processing biomaterials and enabling multi-scale and multi-material auxetics. An engineering design rational for auxetics-based medical devices is presented with integrative purposes. Finally, key research, development and expected technological breakthroughs are discussed.

## 1. Introduction

Auxetic mechanical metamaterials are characterized by a negative Poisson ratio (NPR) and display an unexpected property of lateral expansion when stretched, and equal and opposing densification when compressed. Auxetic properties are uncommon in nature but can be achieved by designing special microstructures and manufacturing them with varied raw materials. The fact that their properties are more a consequence of their designed geometry than of the bulk or raw material employed for their materialization is the defining feature of metamaterials. Auxetics, being mostly used for mechanical applications, constitute a special group within the growing family of mechanical metamaterials. Pioneering studies in the field described the feasibility of materials with negative Poisson ratio, presented examples of auxetic foams and polymers, described varied geometrical design principles, illustrated different design and manufacturing routes for creating auxetics, and proposed potential industrial applications in fields such as robotics, health, scape and transport, to cite a few [1,2,3,4].

Since work in this field began, auxetics have been considered for different biomedical applications, as some biological tissues have auxetic-like behaviour, in addition to a lightweight structure and morphing properties, which makes auxetic geometry ideal for interacting with the human body.

Previous studies have reviewed auxetics applied to biomedical devices in general [5], such as prosthetics [6], tissue engineering [7] and sports practice [8,9]. However, recent progresses in design, modelling and manufacturing processes are transforming the area of mechanical metamaterials, enabling new ways of creating auxetics, applying them to medical or health related devices, and industrially deploying them.

For this reason, authors consider that an up-to-date comprehensive review dealing with auxetics and their connections to the biomedical industry is necessary and can help to put forward interesting research and application directions for these remarkable materials.

This review stands out for providing a complete and up to date view of auxetics for medical devices, including a focus on prosthetics, orthotics, ergonomic appliances, performance enhancement products, in vitro medical devices for interacting with cells, and advanced medicinal clinical products, especially tissue engineering scaffolds with living cells. To the authors’ best knowledge, this study provides the most complete overviews of biomedical applications for auxetic metamaterials.

In addition, innovative design and simulation approaches for the engineering of auxetic-based products are covered, including varied computer-aided and engineering methods such as topology optimization, conformal lattice design, multi-physical finite-element modelling (FEM), multi-scale simulation procedures, artificial intelligence and machine learning, applied to metamaterials designs and to the computational prediction of new auxetics, among others.

Considering the challenges of interacting with the human body and recapitulating tissue biomechanics, the more relevant manufacturing technologies for prototyping and producing auxetics are analysed, taking into consideration those capable of processing biomaterials and detailing those enabling multi-scale and multi-material auxetics.

All the reviewed studies and authors’ personal experiences lead to a summarized engineering design or development rationale for auxetic-based medical devices, with the wish to support colleagues in the field with the straightforward application of auxetics to healthcare. Finally, key research, development and technological transfer expectable breakthroughs are discussed.

## 2. Overview of Medical Applications for Auxetics

**Figure 1:**
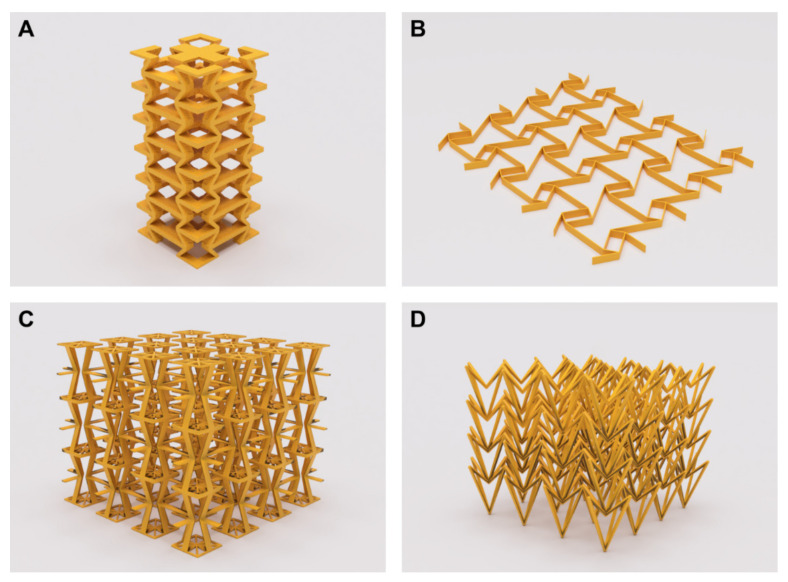
Examples of auxetics that can be used in different medical applications. (**A**,**C**) three-dimensional auxetics based on a re-entrant cell. (**B**) Two-dimensional lattice structure based on a re-entrant cell. (**D**) Three-dimensional auxetic based on an arrow-head unit cell.

Auxetics geometries, as those presented in the library of CAD models from Figure 1, find applications in varied medical technologies, developed in many cases as conceptual alternatives to the *status quo*, but also reaching market and patients as outstanding technological and medical breakthroughs. 

This section reviews some of the most noteworthy examples of biomedical patents, scientific publications, and commercial sanitary products, in which the employment of auxetic geometries provides structural or functional benefits. Subsequently, the study deals with main design, computational modeling and optimization techniques, and a wide set of manufacturing technologies, before proposing a design rationale for auxetics-based medical devices and analyzing current research directions.

The section starts by dealing with auxetic implants for replacing damaged tissues and continues with auxetic orthoses and ergonomic appliances for health and sports related issues. Then, the more innovative research areas of tissue engineering and regenerative medicine, in which auxetics and auxetic scaffolds play innovative roles for interacting with cells and fostering positive mechanobiological responses, are considered. In relation to tissue engineering, in vitro medical devices for cell culture benefiting from the incorporation of auxetic components or subsystems are finally presented. Some connections with the emergent area of biofabrication are made.

### 2.1. Bioprostheses

#### 2.1.1. Spinal Surgery

Artificial intervertebral discs made of high-density auxetic polyethylene can bend and twist and may provide improved biomechanical performance compared with traditional disc replacement solutions. Thanks to its anisotropic negative Poisson’s ratio, the disc prevents bulges that could injure the surrounding nerve endings. Importantly, the disc perfectly mimics the behaviour of a natural lumbar intervertebral disc [10]. Later, Baker put forward a theory on the use of auxetic foams as a material for an artificial intervertebral disc. Auxetic foam has a re-entrant cellular structure with a negative Poisson’s coefficient after heating by triaxial compression. Finite element analysis showed that the use of an artificial intervertebral disc with a negative Poisson’s ratio would be a solution to the problem, as damage to the surrounding nerves by the intervertebral disc is eliminated [11].

As another application linked to spinal surgery, Yan Yao et al. proposed an auxetic pedicle screw based on a Ti6Al4V resin cell (DPR New Materials Technology Co., Ltd., Beijing, China) to improve the biomechanical interaction between the surrounding bone and the screw, mainly for the spine. According to the results of the finite element method (FEM), the correspondence between Young’s modulus of bone and screw is a necessary condition of pull-out protection for a particular bone [12].

#### 2.1.2. Stents

As is well known, a stent is a medical device consisting of a mesh structure, which, when implanted, acts as a framework and supports the patency of the stenosed artery. At the same time, one of the main requirements for stents is high mechanical characteristics of the stent in tension, compression, bending and torsion. This is necessary for the functional reliability of the stent, such as structural support, blood flow regulation, and elevation, etc.

One of such studies [13] was devoted to the development of a new auxetic geometry coronary stent based on 316L medical grade stainless steel, manufactured by laser cutting. The peculiarity of this design allows the stent to retain a certain lumen volume due to simultaneous expansion in two directions under the action of the balloon being inflated. The results of stent diameter and length analysis before and after tension showed that the auxetic coronary stent expanded both in radial and longitudinal directions. In addition, the elastic recoil test revealed no damage to the stent. Auxetic coronary stent can be optimized for a specific diameter and vessel length to create a certain lumen volume, possibly minimizing the negative impact of the stent on the vessel wall.

Liua et al. also conducted an extensive study of auxetic tracheal stents made of soft low-modulus silicone, including mechanical and biological tests. The authors suggest that the cylindrical chiral auxetic hybrid scaffold demonstrates good auxetic properties that can increase the cross-sectional area, thereby improving ventilation and the antimigration strength of the stent. In addition, the stent can solve the problem of mucus blockage, as NHBE cells are able to successfully differentiate into ciliated pseudostratified columnar epithelium on the inner wall of the stent [14].

Relying on quantitative finite element analysis (FEA), Wua et al. [15] investigated the mechanical properties of round- and elliptical-node antichiral stents and hierarchical round- and elliptical-node antichiral stents, as well as their interaction with the arterial model. The authors found that the negative Poisson’s coefficient decreases as the number of cells around the circumference of the stent increases. As the number of axial wells increases for elliptical and circular nodal stents, the negative Poisson’s ratio increases; finally, as the radius of the ellipse increases, the negative Poisson’s ratio and auxecticity decrease.

However, the interaction in the stent–plastic–vessel system is more complex; therefore, the influence of geometrical parameters of the hierarchical antichiral stent with elliptical nodes on the mechanical characteristics of the system should be further systematically investigated in the future.

#### 2.1.3. Hip Implant Stems

Kolken et al. [16] demonstrated the concept of so-called meta-implants, which incorporate a combination of auxetic and conventional materials to improve fixation of the hip stem (total hip replacement -THR-), hence increasing implant durability. THR implants have a limited lifespan, with aseptic loosening being the cause of reduced longevity [17,18,19]. The presented hybrid meta-biomaterial exhibits the most consistent bilateral compression along the boundaries of auxetic and non-auxetic cells. Experimental results showed that the presence of the transition region negatively affects the characteristics of meta-biomaterials, making them less resistant to bending. The absence of the transition region increases bending stiffness, resulting in a more uniform distributed expansion.

Another interesting solution was presented in an article by Ghavideknia et al., which considers a honeycomb hip meta-implant with a gradient Poisson’s ratio. The gradient distribution of three-dimensional re-entrant auxetic cells may be a potential solution for reducing micromotion (relative movement of the implant with respect to bone [20]) and reduce stress shielding effects that can lead to bone resorption.

To achieve this goal, the authors obtained analytical relationships for the mechanical properties of the three-dimensional re-entrant cell, developed four types of implants (solid implant; meta-implant with a positive Poisson’s ratio; meta-implant with negative Poisson’s ratio; and meta-implant with gradient cell distribution) and studied them using finite element analysis (FEA) [21].

#### 2.1.4. Fixation for Long Bones

Studies by the research group of Mehmood et al. involved the manufacture of a polyurethane-based auxetic polymeric bone plate, which can be used as an internal fixator for fractures of long bones. The manufactured construction, in contrast to the auxetic implants for the hip stem [22], allows micromovement, which is of great importance in the process of bone healing. In this case, according to references, micromovement is desirable for the formation of callus [23], facilitating the connection of bone fragments [24,25,26,27,28]. The fabrication of the bone auxetic plate was performed using the injection moulding technique discussed by Ali et al. in early work [29]. The authors showed that the auxetic bone plate has a potential use for fixing the bone in cases where protection against stress shielding and the creation of micromotions is required. Arguably, manufacturing these solutions employing resorbable materials may benefit from auxetic behaviour during healing and lead to a natural state after resorption.

#### 2.1.5. Cardiac Patches

Kapnisi et al. [30] developed auxetic conductive cardiac patches for the treatment of myocardial infarction (MI). The composite consists of an interconnected network of polyaniline and phytic acid grown on the surface of chitosan. The study by [30] has shown that the re-entrant auxetic structure allows the design of patches with an adjustable range of mechanical strength and anisotropy, in accordance with the natural tissue of the heart. In addition, auxetic patches are conductive and have cytocompatibility with neonatal mouse cardiomyocytes in vitro. Auxetic patches maintain a similar level of conduction compared to the previously obtained heart patch without a pattern [31]. Ex vivo studies demonstrate that auxetic patches do not adversely affect the electrophysiology of both the healthy heart and the heart of rats with myocardial infarction and better match each other. Finally, the auxetic patch used in the rat myocardial infarction model had no deleterious effects on cardiac function, and there was no fibrotic response after two weeks of in vivo testing [30].

#### 2.1.6. Nasopharyngeal Swabs

Of note is the article by Arjunan et al. [32]. Although the focus of the paper was the development of an optimal auxetic head for nasopharyngeal swabs, the design concept and analysis methods allow further development of new biomedical products using auxetic agents that can be transferred to digital format and manufactured on demand in a complex current epidemiological situation. The research group developed a nasopharyngeal swab with an auxetic effect that can be produced by 3D printing. It has been suggested that the use of auxetic structures could potentially reduce the stress on the surrounding tissues in the nasal cavity during tampon extraction. The study examined and tested four different auxetic grids and one conventional grid. Parametric analysis showed that the effectiveness of the chosen design depended on the geometric [32].

### 2.2. Orthoses

#### 2.2.1. Orthoses, Bandages, Orthopaedic Insoles

Panico et al. [33] developed a new fractal auxetic element for a conceptual model of an orthopaedic cervical brace. Taking a concave polygon as the basis, using iterative transitions taken from the Koch fractal curve model [34], a new cell was obtained. To provide maximum support to the neck, the geometry of the collar should correspond to the so-called pain map of the patient. The basic idea of the conceptual model is that the “pain map” determines the design of the collar according to the anatomical and physiological needs of the patient, providing flexibility and support according to the user’s conditions. If the neck is flexed, causing the muscles to contract on the one hand and stretch on the other, the auxetic structure can respond in a similar way, in other words, to contract and expand in different parts of it. Thus, according to the authors, the auxetic neck collar is characterized as a personalized product that can be used in everyday active life.

Another line of research was highlighted in a report by Hinrichs et al. [35]. Her team proposed a device that is a shoe insert that promotes healing and protection of the Achilles tendon. The heel insert consists of a multi-layer polyurethane-based auxetic structure (re-entrant auxetic cell) coated with foam, with additional ankle straighteners on the sides. The authors hypothesize that self-contained auxetic support will reduce pain and accelerate healing in patients with Achilles’ tendon injuries and reduce the likelihood of re-injury through additional support.

In turn, engineers at the Massachusetts Institute of Technology [36] have developed flexible mesh materials using a 3D printing method that they can customize for flexibility and strength to simulate and support muscles and tendons. As a conceptual model, the team printed a flexible TPU ankle mesh. The mesh structure was adapted to prevent inward rotation of the ankle, while allowing the joint to move freely in other directions. The mesh was found to increase ankle stiffness during inversion but did not affect it during movement in other directions. Researchers have also developed an auxetic knee brace design that can fit around the knee, even in a bent position.

In the field of cut-offs, particularly orthoses for correcting head deformities, there is an invention patent [37]. It is intended for infants and young children with plagiocephaly, or brachycephaly etc. The purpose of the presented invention is to eliminate some of the disadvantages of existing orthoses, as well as to create an economical, effective, and hygienic orthosis for correcting head deformities.

According to the patent, the orthosis contains a mesh lining (based on a two-dimensional or three-dimensional auxetic structure) with areas of varying stiffness to provide variable pressure to stimulate the growth of certain areas of the head and at the same time restrain other areas. For this, regions of different rigidity contain at least one region of increased flexibility. Enhanced ventilation created by the multiple vents located on the surface of the outer layer will reduce the degree of overheating of the baby’s head. According to a second aspect of the invention, it describes a method for making a head deformity correction orthosis comprising forming a mesh layer, wherein regions of varying stiffness in the mesh layer are adapted to apply variable pressure to the user’s head, to limit unwanted growth and allow for desired growth to correct the deformities.

#### 2.2.2. Sport Protection

Moroney et al. [38] conducted a preliminary study to analyse the functional benefits of auxetic foam in the creation of elements of sports protection. The authors argue that there is an opportunity to explore the potential of auxetic materials to improve the functionality of protective elements through synclastic curvature and biaxial expansion. For this purpose, authors studied the effect of stretching and curvature of the body on the size of auxetic and regular foam attached to the shoulder area of a sports top. The results of the study indicated that auxetic foams have the potential to be further developed as PPE in sportswear and that further research should be developed with a focus on optimal parameters for its application, including bonding and sealing methods.

### 2.3. Tissue Engineering and Biofabrication

#### 2.3.1. Scaffolds

Scaffolds, according to the complex of mechanical properties, microstructure, bioactivity, chemical composition, among others, affect the behaviour of cells/implant interaction. Chen et al. [39] suggested auxetic hydrogel scaffolds based on fish gelatine methacrylamide and studied cyclic tensile stimulation effects on the neural differentiation capabilities of human Schwann cells.

Authors found that the tensile forces were able to enhance cell viability and proliferation, and the secretion of neural regeneration-related proteins.

Additionally, Flamourakis et al. [40] suggested adaptable auxetic scaffolds for tissue engineering applications. They showed that mouse fibroblasts can penetrate such structure and proliferate. It should be noted that in some of the works, it is precisely the influence of the pore structure formed during the transition to a material with a negative Poisson’s ratio on the interaction with cells, and not the auxetism during deformation itself that is investigated in detail.

Yan et al. [41] suggested auxetic polyurethane scaffolds and showed that auxetic scaffolds (ν from 0 to −0.45, E from 10 to 100 kPa, pore size range 250–300 μm), in comparison with non-auxetic porous scaffolds, supported smaller aggregate formation and higher expression of neuronal marker β-tubulin III upon neural differentiation of iPSCs.

Later, the same authors [42] demonstrated that auxetic scaffolds were able to transfer the compressive load isotropically to the cells and iPSCs can be influenced to differentiate into vascular lineage cells. Lantada et al. [43] studied cell attachment and viability on auxetic 2D scaffolds, whose biomimetic geometries may have a relevant role in vascularization processes [44]. Microstructure of 2D scaffolds can be more precisely controlled (see Section 4), but with further limitations in applications, when compared to 3D scaffolding structures.

A combination of materials with negative and positive Poisson’s ratio in a single medical design can be promising from the point of view of tissue engineering and biomimetics. Soman et al. [45] fabricated a PEG scaffold, which exhibits simultaneous negative and positive Poisson’s ratio behaviour. Such structures may be more suitable for emulating the behaviour of native tissue mechanics.

#### 2.3.2. Auxetic Structures and Membranes for In Vitro Medical Devices

Auxetics have been reported to mimic the mechanical properties of human tissues and their use in implantable devices and tissue engineering has been reviewed in previous sections. However, these biomechanical properties also show benefits from a mechanobiological perspective for cell culture systems and microfluidics. For example, the nuclei of embryonic stem cells, during their transition state, have been reported to have auxetic properties [46]. Arguably, auxeticity can be a relevant element in mechanotransduction, as authors from that study proposed. Using auxetic structures for such in vitro studies and a better understanding of angiogenesis is a promising research direction. However, to interact in vitro at a single cellular level, special micro-/nano-manufacturing technologies may be needed [47], as described in the section dealing with prototyping and manufacturing methods.

Moreover, different commercial devices for in vitro studies have been developed, which include auxetic elements or geometries within, such as the bioreactors with auxetic membranes for biaxial stimulation of cell cultures developed by CellScale [48]. The incorporation of such bioreactors with alternative membranes with regional auxetic properties [49] can be of interest for cell co-culture and mechanobiological studies, especially for in vitro studying of the transition between auxetic tissues such as tendon and non-auxetic tissues, e.g., bone.

## 3. Design and Modelling of Auxetic Geometries and Implants

**Figure 2:**
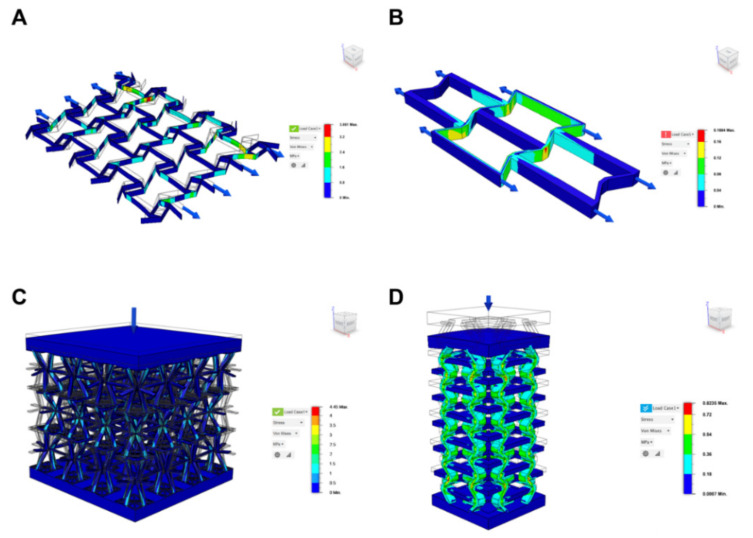
Examples of computational modelling applied to auxetic metamaterials. FEM modelling of displacements of auxetic cells under quasi-static loading. (**A**,**B**) Two-dimensional lattice structure based on re-entrant cell. (**C**,**D**) Three-dimensional auxetic based on re-entrant cells. Computational modelling was performed using Fusion Autodesk 360 (Santa Monica, CA, USA) software specifically for the article.

A wide set of computational methods have helped to develop the area of auxetic metamaterials to promote their application to biomedical devices, and to accelerate the discovery of new auxetic geometries. This section summarizes such computational methods applied to biomedical auxetics, from the more conventional-like state-of-the-art computer-aided design (CAD) resources and finite element modelling (FEM) tools, as shown in Figure 2, to recent artificial intelligence (AI) and machine learning (ML) aided design and quasi autonomous search for innovative metamaterials with atypical Poisson ratios.

These resources help to characterize cell units, which can subsequently be used to construct the structures of more complex medical devices, by application of Boolean and matrix-based operations, or following conformal lattice design approaches which promote personalization, if the design starts from medical images or three-dimensional patients’ geometries reconstructions. Moreover, these resources support designers in the optimization of geometries, materials, and processes. They help with the in silico evaluation of final devices’ performance, including evaluations of their operational limits.

### 3.1. Supporting Simulation and Topology Optimization Resources

Auricchio et al. [50] focused on the idea of layered support materials based on the existing tetrachiral honeycomb structure. Solid state finite element analysis (FEA) of both models was performed to predict their deformation behaviour and to confirm experimental measurements of tetrachiral displacement under uniaxial tensile loads. The authors also used a low-dimensional Lagrangian formulation within linear mechanics, called the “beam lattice model” as an alternative to FEA, which is associated with large computational burdens. The resulting bitetrachiral samples exhibited the kinematic elimination of angular deformation characteristic of chiral auxetics, as well as a global Poisson’s ratio close to −0.7 and a global Young’s modulus superior to that of the monotetrachiral structure. The physical test results were validated qualitatively and quantitatively using the FEA model and the beam lattice model. The authors note that an overall Poisson’s ratio as low as −1 can be achieved by changing the unit cell parameters.

In their work, Wilt et al. [51] used deep learning technologies to create a workflow for designing two-dimensional auxetic metamaterials. The authors argue that complex analytical equations combined with traditional control algorithms are often used to control malleable gradient mechanisms, including auxetics, making their design difficult. The proposed workflow, based on finite element analysis (FEA) and convolutional neural networks (CNNs), is designed to reduce the computational burden of auxiliary material design by predicting possible flaws and errors based on a mathematically optimal deformation concept. CNNs are trained using pseudorandomized images and corresponding FEA results, resulting in a regression model capable of calculating differences between the desired and actual deformation behaviour of samples.

### 3.2. Computational Prediction and AI-Aided Design of New Auxetic Geometries

Nguyen et al. [52] presented geometric construction methods for complex shaped cellular structures called Conformal Lattice Structures™ (CLS). These structures consist of non-uniform lattices aligned to surface parts. The CLS design pipeline begins by acquiring a computer-aided design (CAD) model of an original part and desired element size as an input parameter. The part boundary is divided into relatively flat regions by comparison with normal neighbouring surfaces. Each region then undergoes a three-stage process of computing object boundary offsets, constructing a lofted volume between original and offset surfaces, and parametrically subdividing that volume into individual elements (hexahedra) to form a mesh. Mesh-formed regions are merged with the addition or removal of elements. The resulting parametrized mesh is then populated with unit cells selected from the existing library.

Variations of the conformal lattice design are currently being studied and are becoming popular approaches linked to designs for additive manufacturing strategies [53,54,55]. One remarkable study focused on the augmentation of the Size Matching and Scaling (SMS) method to better suit the production of meso-scale conformal lattice structures (MSLS) through the principle of stress distribution similarity between the solid body and CLS of the same shape [53]. Design of MSLS also considers different configurations of each unit cell during mesh population due to their selection criteria, so that the cells may be parametrically mapped into existing hexahedral mesh elements. Different algorithms may help to control desired stiffness [54].

Final pre-production steps can include optimization to reduce weight or increase mechanical properties of MSLS [55] and converting a CAD file into STL format [56], which is standard amongst Additive Manufacturing methods. Generally, the presented “free-mesh” approach decreases the computational load during MSLS design process by reducing multivariable optimization problems to a problem of two variables: solid-body analysis and predefined unit-cell library. In this case, conventionally used rigorous topology optimization is considered a main bottleneck in designing MSLS, although related multi-scale manufacturing challenges should not be discarded either (see Section 4).

Dagdelen et al. [57] studied computational prediction of inorganic crystalline auxetic materials by means of screening and deriving each suitable material’s Poisson ratio from its pre-calculated elastic sensor. The authors summarize that the accelerated discovery of novel materials with target properties is desirable to meet current and future material needs. They show how the discovery of materials with unusual elastic behaviour can be further accelerated by means of tiered search, targeting specific structural motifs. Research described in the article proposes three new homogenous auxetics through the hypothesis that the structural motif—encompassed by rigid, corner-connected tetrahedra and open structure—is the dominating driver for auxetic behaviour.

## 4. Prototyping and Manufacturing Methods

The processing of biomedical alloys, ceramics, polymers, and biomaterials in general, and their structuring with auxetic topologies is achieved using several manufacturing technologies including well-established mass-production tools such as rolling, stamping, foaming and injection moulding. Rapid prototyping and freeform fabrication tools, such as fused-deposition modelling, selective laser sintering and melting or additive photopolymerizations and rapid tooling approaches, towards high-end research-oriented systems for the creation of metamaterials, such as two-photon polymerization, to cite a few. 

The main techniques for the materialization of auxetic geometries and their biomedical applications are presented below.

### 4.1. Traditional Methods

Currently, mass-produced medical devices rely on more traditional technologies than AMTs, such as injection moulding, compression moulding, stamping, rolling, foaming, CNC machining or textile-based processes, and this applies also to several examples of biomedical auxetics. Mould-related procedures typically apply to mass-produced polymeric auxetics. Foaming has been applied to the creation of auxetics since early endeavours in the field [1] and is still a useful, straightforward, and mass-production procedure [58], although morphological and structural control from the design stage is challenging and relies on human experience, compared to additively manufactured auxetics.

More recently, injection moulding of rate stiffening re-entrant cell arrays have proven useful for wearable impact protection [59] and the procedure is clearly oriented to mass-production and industrial impact promotion, although limited in terms of geometrical complexity when compared to layer-by-layer methods. This additional level of complexity may be achieved by stacking different injection moulded auxetic sheets, which may even lead to functionally graded constructs, potentially applicable to mimicking the typical gradient properties from human tissues. For high-performance auxetic alloys, the lost-wax casting process is usable [60], as well as CNC machining [61], which nevertheless is limited in terms of geometrical complexity.

#### 4.1.1. Rolling, Casting, and Foaming Methods

The article by Grujicicic et al. [62] considers processes of obtaining metallic sandwich structures with an auxetic hexagonal core. Using various analytical methods, the influence of these processes on the grain microstructure of metal workpieces and response of sandwich structures to explosive loading were investigated. The authors analysed three obtaining ways: a sheet metal fabrication using cold flat rolling, a stamping process, and laser welding. It was pointed out that each process has a different effect on the microstructure of grains and on the crystallographic texture of workpieces. However, the paper does not evaluate the effect of the negative Poisson’s ratio of the sandwich structure on the results of blast loading.

One of the methods of obtaining auxetic structures is polymer casting. The research of Ali et al. [29] focused on creating a suture-less auxetic oesophageal stent whose design was to facilitate oral insertion of the stent into the body and improve its mechanical properties. For this purpose, a collapsible tubular matrix made of different materials: ABS plastic, titanium alloy and special reinforced glass was designed. This matrix consisted of two halves that had the same auxetic pattern on the inner sides—alternately multidirectional rhombuses. After that, the matrices were connected around a Teflon rod, in which the polyurethane was then poured. The latter was performed using compressed air, so that the polymer would fill the entire space. The structure was left to cure for 12 h, after which the matrices were split in half and the Teflon rod was removed. The effectiveness, speed, and simplicity of this method were analysed by comparing it with other methods. One such method is casting polyurethane onto special substrates made of different materials: ABS plastic; ABS coated with a nickel-phosphorus alloy; and titanium alloy. Such substrates have a relief inverse to the auxetic pattern of the films obtained.

Another example of the production of auxetic foams is demonstrated in work fabrication and characterization of auxetic shape memory composite foams. Scarpa et al. [63] proposed a shape memory composite foam based on polyurethane (PU) foam as a matrix and epoxy resin with shape memory (Er) as a dispersed phase. The authors obtained auxetic PU/Er foams through a triaxial compression process followed by heat treatment. The auxetic foams showed an increase in stiffness over conventional foam. Furthermore, it recovers their shape better as the amount of resin increases.

#### 4.1.2. Textile Based Processes

Jiang et al. [64] presented a method of obtaining an auxetic braided structure based on the spiral principle. The whole process of creating an auxetic structure includes two steps: formation of the basic braided structure and fixation of the wrapped yarn. To do this, the yarn is spirally wound around the tubular braid and secured at four equally distant points in each repeating turn of the spiral. The fixation ensures uniform winding of the wrapping thread on the outside of the braid. Additionally, it reduces the risk of slippage that can occur in an auxetic spiral thread. The winding angle, weave angle, and diameter of the braided yarn are parameters that affect the mechanical properties of the final auxetic braided structure. It is reported that the winding angle has a greater influence than the other two parameters [64].

Another method of making textile auxetic structures is helical auxetic yarn (HAY). The HAY method is based on the spiral winding of a stiff yarn on a bobbin. A special “spinner” was designed by the authors to create the spiral yarn [65]. The fibres were wound on spools by hand to ensure a uniform winding of the fibre with minimal tension. Thus, the paper by Bhattacharya et al. [66] investigated the effect of the interaction of stiff yarn and fibre in the core on the Poisson’s ratio of the resulting yarn. It was shown that the effect of pressing the outer yarn into the core has a negative effect on the final auxetic behaviour of the yarn. It was also observed that the negative Poisson’s ratio increases if the stiffness of the outer yarn is higher than the stiffness of the core, provided that no indentation occurs [66].

Finally, Zhou et al. [67] developed a three-dimensional (3D) auxetic textile structure based on polyurethane (PA) foam. The results showed that a negative Poisson’s ratio of composites can be obtained by using a suitable yarn textile structure. The structure found an application as a reinforcing component for the fabrication of auxetic composites. The authors compared the fabricated auxetic composites with non-auxetic composites made from the same materials. They found that the auxetic and non-auxetic composites had different mechanical behaviour: while the auxetic composites behaved as a cushioning material at lower compressive stress, the non-auxetic composite behaved as a stiffer material at higher compressive stress. It was shown that using auxetic fibres as reinforcement demonstrates better mechanical properties compared to other auxetic structures. The reason is the increase in strength of the matrix–fibre interface, which allows the composites to withstand twice the maximum load compared to other auxetic structure variants [67].

### 4.2. Additive Manufacturing

Mechanical metamaterials have emerged in recent decades [68,69,70], to a great extent, thanks to parallel progresses in additive manufacturing technologies (AMTs). These resources, generally working on a layer-by-layer approach, enable solid freeform fabrication and the creation of extremely complex devices, structures, and materials. Raw materials, including polymers, ceramics, alloys, composites, and even biological materials and living cells, can be additively processed, and many are adequate for interacting with human tissues and developing biocompatible medical appliances [71].

Moreover, the resolution, precision, and achievable printing sizes, among other relevant features of AMTs, have been continuously improving. Nowadays, it is possible to achieve structures with submicrometric precision for interacting at a cellular level [72], as well as high performance structures for repairing large tissues [73]. 

Auxetics, as a relevant group within the growing family of mechanical metamaterials, also benefit from being additively manufactured and many 3D auxetics are only achievable by means of additive procedures, as recently reviewed [74], or employing procedures involving additively manufactured tools.

Polymeric 3D auxetics have been achieved, for example, using fused-deposition modelling (FDM) [75], which is low-cost and highly interesting for biomedical applications as several PLA, PCL and TPU medical grade printing filaments are already available. 

However, the achievable precision and complexity with FDM do not match those of additive photolithographic or stereolithographic systems, which have also been applied to the manufacture of complex auxetic geometries [76].

Notwithstanding the benefits in terms of precision of photopolymerization systems, a common limitation of stereolithographic systems for the biomedical field is the lack of compatibility of conventional photoresins, although recent advances in biophotopolymers are progressively solving this drawback, as has already been demonstrated with auxetic structures manufactured by dynamic optical projection stereolithography (DOPsL) of tissue scaffolds using biomaterials [49]. Selective laser sintering of polyamide auxetic stents have demonstrated the interesting features of this technique for the field of biomedical auxetics [77]. It stands out for the possibility of manufacturing supportless large structures, although its surface properties and resolution still do not match those achievable through additive photopolymerization (i.e., stereolithography or digital light processing).

Regarding high-performance materials for hard tissue repairs, i.e., alloys and ceramics, different additive manufacturing technologies can be also applied to the area of biomedical auxetics. The applicability of selective laser melting to the creation of Ti-6Al-4V auxetic meta-biomaterials has been detailed and their mechanical properties analysed [78]. In addition, Xue Y. et al. [79] developed a technology by combining the 3D printing technique with investment casting to solve the problem of metallurgical defects during 3D printing (especially if the printing material is chemically active, as with Al and Mg alloys). In this method, an auxetic lattice structure is made from a light-sensitive resin using 3D printing. After that, the structure is used as an aluminium casting mould followed by pressure infiltration to produce a honeycomb structure. This technique reduces the occurrence of metallurgical defects. The resulting aluminium-based auxetic lattice structures have strength characteristics. This is an alternative way of making auxetic or other metal-based lattice structures based on 3D printing technologies [79].

Taking ceramic materials into account, lithography-based ceramic manufacturing [80], industrially developed by Lithoz GmbH, stands out for its precision, resolution, and diversity of ceramic materials available, which benefits auxetic metamaterials [81] and their future biomedical applications. Modifications of this technology are leading to multi-material constructs and to lithography-based metal manufacturing.

In the area of composites, 3D printing-directed auxetic Kevlar aerogel architectures have been also achieved by developing and applying an innovative combination of direct ink writing (DIW) and freeze-casting with dimethyl sulfoxide (DMSO)-based inks [82].

Considering smart materials, a study by Tang, H. et al. [83] showed that the combination of an auxetic structure and piezoelectric ceramics can lead to auxetic piezoelectric meta-materials that combine the respective advantages of auxetic and piezoelectric materials. The authors verified that piezoelectric ceramics with ultra-low porosity can have good mechanical performance based on the concept of auxetic piezoceramics. Due to the ultra-low porosity and the auxetic effect, the meta-material should have higher stiffness and crack resistance. It should also have higher impact toughness and be easier to polarize [83].

**Figure 3:**
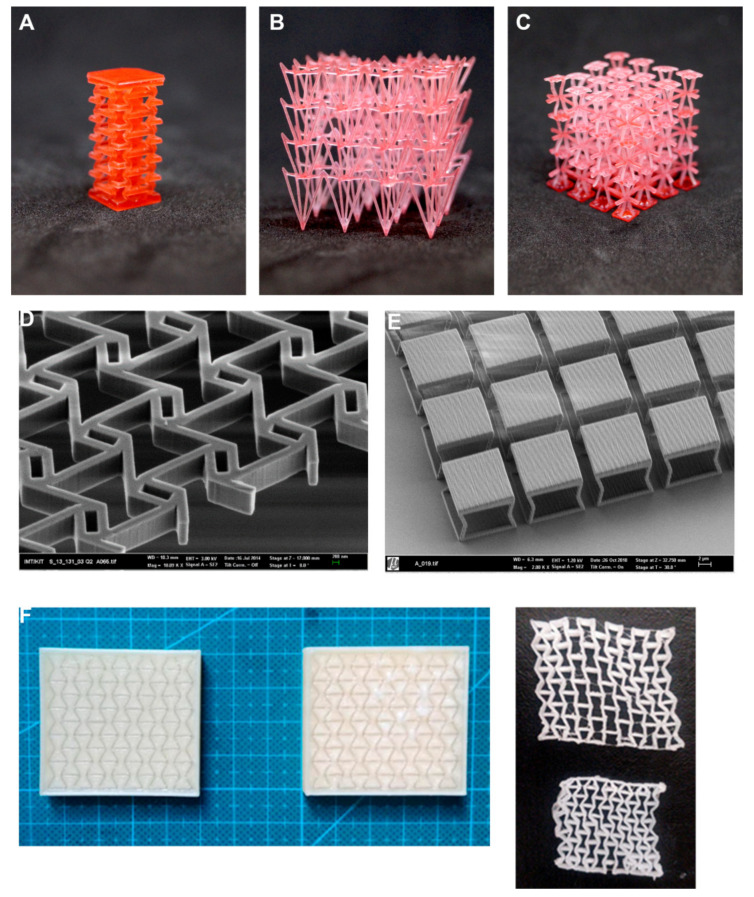
Examples of auxetics manufactured with different technologies and materials. (**A**–**C**) Three-dimensional auxetic made by laser stereolithography. (**D**) Deep reactive ion etching of silicon [43]. (**E**) Two-photo polymerization of metasurface with pixels supported by re-entrant pillars. Examples (**A**–**C**) were performed using Phrozen Transform Standard (Phrozen Ltd., Hsinchu, Taiwan) with HARZ Labs Basic Red resin (Moscow, Russia) specifically for the article. Examples D, E are manufactured with the support of the “Karlsruhe Nano Micro Facility, a Helmholtz Research Infrastructure” (for additional details, please check: [43,86,87]). (**F**) Soluble 3D printed PVA moulds and PDMS auxetic structures obtained by casting and mould dissolution, as indirect procedure to obtain auxetics (courtesy of Adrián Martínez Cendrero).

Indirect methods, employing additively manufactured moulds, tools or inserts, and the progress with soluble resins and thermoplastics for the 3D printing field may also prove interesting for the development of biomedical auxetics and help to enhance the variability of processable biomaterials. For instance, as illustrated in Figure 3F, a soluble PVA mould can be printed, leaving a hollow space with auxetic geometry. The casting of a PDMS and the subsequent dissolution of the mould led to a final flexible auxetic made of a bulk material common for biomedical appliances. The employment of inserts in such soluble moulds may straightforwardly lead to multi-material constructs for tissue repair, which becomes especially useful for complex restorations, in which different hard and soft tissues should be jointly reconstructed.

Despite the evident impact of AMTs on the scientific-technological expansion and industrial deployment of auxetics, especially for the biomedical arena, it is important to mention that other traditional mass-production techniques are still unrivalled when it comes to mass-production of simpler 2D auxetic structures, as detailed below. 

Furthermore, expectable progress in the manufacturing speed and processability of medical grade materials will surely reinforce AMTs as technologies of choice for biomedical auxetics in parallel to advances in personalized healthcare.

Both traditional mass-production technologies and conventional AMTs may be limited in terms of manufacturing precision, especially for the development of bio-MEMS aimed at interacting at cellular or even molecular levels. To this end, technologies from the electronic industry and from the realm of photonics are applicable, as well as other well-established micro/nanomanufacturing methods described further on.

### 4.3. Micro/Nanomanufacturing Methods, Multi-Scale and Multi-Material Approaches

UV photolithography, common technology for micropatterning and surface micromachining, is applicable to the manufacture of 2D or 2D ½ auxetic geometries [84] and may apply to a wide set of photopolymeric films and etchable semimetal (i.e., Si) and metal sheets (Cu, steel…). 

Soft-lithography, a technique that employs photopolymerized patterns (obtained by UV photolithography) as a tool for creating soft PDMS moulds, stamps, and sheets, is a low-cost rapid-prototyping tool for creating 2D auxetics in medical grade materials. By rolling, these can be transformed into stents and other 3D medical devices [85]. Deep-reactive ion etching, an improved UV lithography related method, has led to some of the most precise microstructures for interacting at a single cellular level [86]. In any case, these techniques are mostly applicable to quasi two-dimensional bio-MEMS, as happens with most technologies evolved from the microelectronics industry.

More geometrical complexity and three-dimensional structures for micro-auxetics are achievable through multi-photon [40] and two-photon [87] polymerization. In the case of two-photon polymerization (2PP) details down to a few hundreds of nanometres are achievable. Considering recent advances in biomedical photopolymers for 2PP and the processability of multi-material structures, this technique is a fundamental breakthrough for auxetics and metamaterials in general.

Multi-material auxetic cells with enhanced actuation ability have also been demonstrated using the poly-jet technology of Stratasys [88], characterized by a jetting head capable of depositing inks of multiple polymers, some of them of medical grade. The remarkable precision of the technology and the usability of multiple materials promotes multi-material and multi-scale approaches [89], which are extremely relevant for the future of mechanical metamaterials, especially if biomedical applications are pursued.

Apart from these high-precision AMTs, other options such as electrospinning [90] and multi-scale melt electro writing [91] have been applied to the creation of auxetic porous geometries with potential applications in tissue engineering.

In the authors’ perspective, further research aimed at integrating the technologies and materials is required for reaching truly high-performance, multi-scale and multi-material auxetics production lines for biomedical applications.

## 5. Summary of Design Rationale and Development

The reviewed solutions not only demonstrate the relevance of auxetics for innovation in the medical field and reinventing several existing medical devices, but also help to put forward a design rationale for choosing auxetic geometries for biodevices, together with a development workflow, in which key questions, control points and decisions are mapped.

This workflow may become a systematic development methodology in the future, once validated through additional cases designed, manufactured and tested following the proposed approach.

In the conceptual design stage, the driving questions could be:

(1) Are solutions already employed for solving the concrete optimal medical needs?
(2) Would an auxetic geometry provide potential benefits?

Reviewed examples have shown that biomedical devices are often suboptimal in terms of biomechanical behaviour, long-term performance, ergonomics and even aesthetics. The use of auxetics in the medical field has already demonstrated benefits linked to biomimetic behaviour, enhanced adhesion or interaction with human tissues, improved visual appeal and differentiation from already patented solutions, to cite a few. These reasons could be enough to initiate any redesign of an existing medical device with an innovative auxetic perspective.

Considering the basic engineering stage, which deals with geometries, materials and prototyping tools, relevant questions include:

(3) Is it possible to transform state-of-the-art solutions into innovative auxetic-based designs?
(4) Should the design be oriented to personalization or mass production?

**Figure 4:**
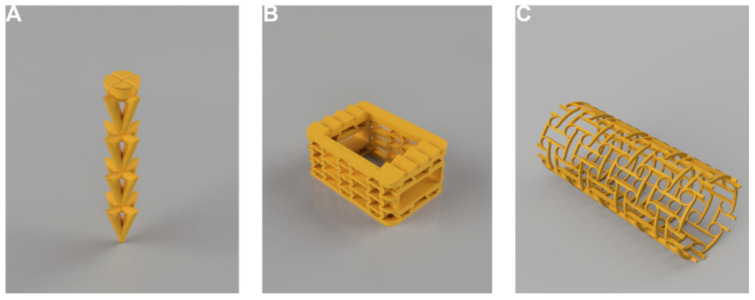
Examples of CAD models of different medical devices benefiting from employing auxetic units or having an auxetic geometry. (**A**) Auxetic insert for enhanced surgical fixation. (**B**) Spine disc replacement made of auxetic lattices for biomimetic performance. (**C**) 2D auxetic geometry mapped upon a cylinder to obtain an auxetic stent with improved implantation and adhesion.

**Figure 5:**
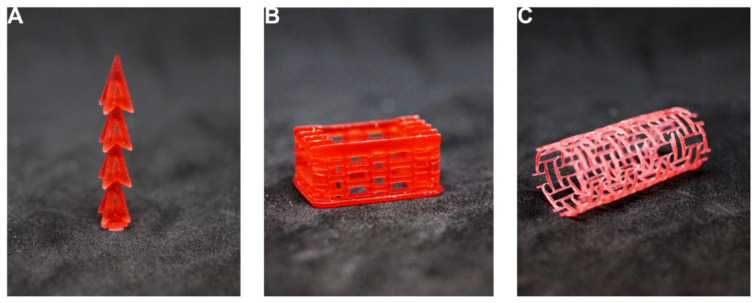
Examples of related prototypes of different medical devices benefiting from employing auxetic units or having an auxetic geometry. (**A**) Auxetic insert for enhanced surgical fixation. (**B**) Spine disc replacement made of auxetic lattices for biomimetic performance. (**C**) 2D auxetic geometry mapped upon a cylinder to obtain an auxetic stent with improved implantation and adhesion.

Depending on the actual medical device and biological structure(s) with which the auxetic structure will interact (or replace), the final geometries will be more or less challenging to conceive and design. However, existing examples demonstrate that auxetic-based designs are always achievable, either using simple CAD tools such as Boolean operations and matrix-based replications or resorting to more complex topology optimizations and tools for mapping 2D and 3D lattices within CAD models (see examples from Figure 4 and Figure 5). Regarding manufacturability, freedom of design has been importantly promoted through additive manufacturing tools, so any design may be “printable” in a wide set of materials and employed in varied technologies, many of them very affordable. For a personalised design, a combination of medical images (MI) with CAD modelling is already state-of-the-art, and rapid prototyping is also directed through conversion modules that most MI-related software already incorporates.

Taking account of the actual testing and validation stages for the detailed or embodiment engineering and production planning phases, key questions involve: (5) What resources can help optimize the basic design into a final product? (6) Are the production technologies and materials different from the prototyping ones?

Geometries, materials, and production technologies should be jointly considered when planning the optimization of biomechanical devices and approaching patients. Additive tools are progressively employed both for prototyping and for production steps. Moreover, considering optimization and its connections with certification, computational tools provide an increasingly large collection of methods for the in-silico evaluation and optimization of medical devices. Finite-element modelling, mesh-free strategies, topological optimizations, AI/ML approaches, etc. have shown important efficiency-related benefits for design and manufacturing optimization purposes. The intricate geometries of auxetic lattices, if not adequately optimized, may lead to stress-concentration problems and challenging issues in regard to fatigue behaviour. The systematic employment of computational tools, together with prototyping and testing methods, acquires special relevance, as is directly connected to final device safety.

In all cases, together with these specific questions dealing with the actual challenges of defining, manufacturing, and testing auxetic geometries and applying them to medical devices, the required ethical, legal, safety and social aspects applicable to sanitary products in general should be considered.

A possible development rationale based on the above considerations is schematically presented in Table 1 with a focus on personalized medicine and with selected good practices for the different steps.

## 6. Future Trends and Challenges

**Figure 6:**
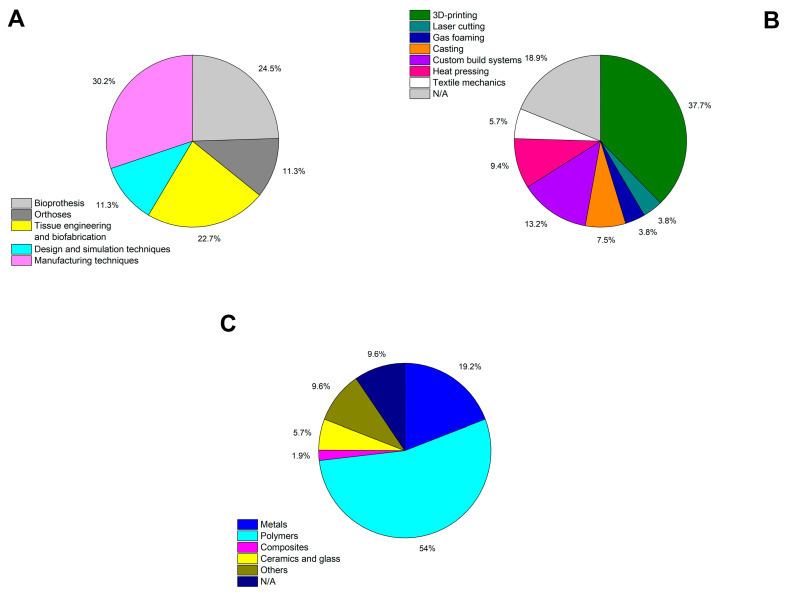
Summarizing statistics of the works reviewed in the current article: (**A**) by type of study; (**B**) by method of production; (**C**) by materials. The summary information for each examined paper can be found in Appendix A.

Auxetics are progressively finding applications in the biomedical field, as has been reviewed (and is schematically summarized in Figure 6 and Table A1). However, in most cases these are still conceptual prototypes needing additional research for successfully reaching patients. Several challenges still need to be addressed: 

Many complex structures of 3D auxetics can only be achieved by additive manufacturing procedures. Although these technologies have radically evolved since the beginning of 21st century, entering the biomedical industry and offering wider sets of medical grade materials and improvements in terms of precision and performance, there still remain questions that need to be answered, especially regarding the long-term performance of auxetic-based medical devices. For example, the fatigue behaviour of auxetic lattices [92] should be considered and additionally studied, especially for powder-based AM processes, in which surface flaws are common. Moreover, most additive technologies lead to intrinsically anisotropic results, due to the layer-by-layer process, which may affect mechanical performance, an influence that is difficult to predict from the design stage. Finally, printing with “smart” alloys, such as shape-memory alloys and biodegradable alloys, which could lead to auxetic-based implants with shape-morphing properties for minimal invasiveness, or geometrical evolutions to accompany patients’ healing and growth, is not yet well established.

Regarding the range of applications, auxetics for sports and orthoses are already well covered and lead to remarkable results, in addition to auxetics for research in the tissue engineering field, with several examples of useful in vitro models. However, we believe that the most transformative clinical impacts can be achieved by further exploring the potential benefits of auxetic structures for medical instruments and for implantable devices (bioprostheses). Indeed, the expandable structures of auxetics, if adequately designed, documented and shared [93], translated to the geometries (both simple and complex) of varied medical instruments and manufactured with the adequate materials and technologies, can foster minimally invasive procedures in varied clinical exploration and surgical procedures. For example, radially expandable auxetic tubes could be applied as structural elements for enhancing the performance of vaginal speculum in order to avoid the common uncomfortable pinching of the usual two blade design. Endoscopes and colonoscopes with auxetic geometries could be envisioned, based on designs applied to tubular oesophageal and tracheal auxetic stents.

Considering implants, metamaterials combining conventional and auxetic lattices could be designed to achieve functionally graded materials, as needed for complex restorations, in which varied tissues with graded mechanical properties are involved. Spinal disc replacement devices and osteochondral plugs, in which the densifying properties of auxetics under compression may help better mimic the properties of cartilage, would be candidates for such graded lattices, as we intend to explore in following studies, especially by using multi-material and multi-scale printing of alloys and polymers.

Together with the mentioned challenges, some emergent research trends can help further empower auxetics for biomedical applications and beyond. Synergies between 4D printing and auxetic geometries can further promote developments of shape morphing medical devices [94,95]. The manufacturing of shape memory materials and mechanisms can bring auxetic based actuations a step forward, as many auxetic geometries rely on rotating elements. Printing mechanisms simplifies the production of auxetics in a single procedure and directly from the design stage, while using shape memory materials for printing can help control the motility of auxetics and tailor it to specific surgical procedures or to the actual healing process after implantation.

Another emergent field that may benefit from employing auxetic geometries is living materials and machines [96,97], especially biohybrid materials [98], in which an auxetic scaffold colonized by cells can lead to more autonomous actuators, even with self-healing properties, ideally capable of outperforming the abilities of currently available “smart”, active or multifunctional materials and structures. Biohybrid microbots with auxetic chassis and mechanisms operated by living cells, such as cardiomyocytes or musculoskeletal cells activated by electric stimuli, may well be used as surgical actuators in the future.

## Figures and Tables

**Table 1:** Schematic summary of development workflow for biomedical auxetics.

**Patient**	
Evaluation of the medical problem. The medical team with support of biomedical technology designers and developers analyse if a mass-produced medical device or a custom-made solution is advisable. The potential benefits of auxetic geometries for enhanced implantability, improved biomechanical performance, or promoted ergonomics/aesthetics are discussed.	
**Medical Imaging**	
CT/MRI data employed as starting point in DICOM format. Segmentation and processing of the anatomical part of interest. Obtaining a 3D model of the defect or region of interest using “MIMICS-like” software resources (i.e., STL format, as input for CAD modelling and design personalization.For external devices such as orthoses, more affordable and direct optical imaging systems may be employed, even based on smartphones’ cameras and dedicated software, to obtain the geometry.	
**Choice of Auxetic Geometry**	
Selection of auxetic geometries from design libraries based on the type of problem and the behaviour of the different auxetics. Open-source libraries may promote healthcare equity. Library of auxetics forloaded products (scaffolds, artificial disks, etc.), for which re-entrant auxetics may work better.Products requiring flexibility (for example for minimal invasion such as stents, or skin and muscular patches or soft tissue engineering), for which chiral and rotating auxetics may work better.	
**Designing with CAD Systems**	
Creation of a medical device using standards to design an original product. Adaptation of an existing medical device by incorporation of auxetic features or regions. Matching with a 3D model of the defect for personalized approaches.	Designing auxetic geometries is possible in varied ways and through a combination of different strategies: − Creation of a solid form, carving a pattern of auxetic geometry by means of Boolean operations; − Creating a unit cell of auxetic geometry (using Boolean operations, copying the unit cell to the required dimensions. Deformation of the resulting model by bending.
**FEM Simulations and Biomechanical Optimization**	
Simulation of simplified mechanical, thermal, fluidic and other tests to determine compliance with required standards and objectives. Evaluation of biomechanical interaction with the body and verification of improved performance of the auxetic devices, as compared with current gold standards.	
Manufacturing of prototypes (i.e., 3D printing and reviewed tools) for experimental evaluation	
Three-dimensional printing or rapid manufacturing of a prototype employing testing materials or materials used in the manufacture of medical devices allows developers to evaluate the design quality and potentials.Systematic in vitro trials with biomimetic work benches and dummies for checking the simulations and design optimization purposes, testing also the improved designs for safely approaching medical trials.	
**Validation**	
Conducting systematic tests according to internationally recognized standards (ISO 10993 for biocompatibility, ISO 14971 and ISO 13485 for risk and quality management etc.).Quality assessment by the surgical planning team in the case of custom-made or patient-specific solution, CE-marking or similar certification depending on applicable regulations for mass-produced devices.Final creation of the custom-made device or production planning, supply chain management and marketing for mass-produced devices.	

## Data Availability

Data and CAD models of presented auxetics and biomedical devices structures based on them are available on reasonable written request to authors.

## Appendix A

**Table A1:** Summary of studies dealing with auxetics for biomedical applications and their design and manufacturing processes.

	Study	Research Type	Research Objective
1	Martz et al. [10]—Design of an artificial intervertebral disc exhibiting a negative Poisson’s ratio	Spinal surgery	Investigate the feasibility of an artificial intervertebral lumbar disc to eliminate problems of bulge
2	Baker et al. [11]—Auxetic spinal implants: consideration of negative Poisson’s ratio in the design of an artificial intervertebral disc	Spinal surgery	Investigate possibility of eliminating the damage to the surrounding nerves by the artificial intervertebral disc
3	Yao et al. [12] –Biomechanical design and analysis of auxetic pedicle screw to resist loosening	Spinal surgery	Design of auxetic pedicle screw to improve the biomechanical interaction of the surrounding bone and screw
4	Amin et al. [13]—Auxetic coronary stent endoprosthesis: fabrication and structural analysis	Stents	Development of a coronary stent for increasing mechanical adhesion with the arterial wall
5	Liua et al. [14]—A flexible porous chiral auxetic tracheal stent with ciliated epithelium	Stents	Development of a tracheal stent for expanding the ventilation cross-section and enhance the anti-migration force of the stent
6	Wua et al. [15]—Mechanical properties of anti-tetrachiral auxetic stents	Stents	Study of coronary stent interaction with an arterial model
7	Kolken et al. [16]—Rationally designed meta-implants: a combination of auxetic and conventional meta-biomaterials	Hip stems implant (THR)	Development the efficient hybrid implants for increasing implant longevity
8	Ghavidelnia et al. [21]—Femur auxetic Meta-Implants with tuned micromotion distribution	Hip stems implant (THR)	Development of a hip stems implant with gradient auxetic structure to reduce micromotions and stress shielding
9	Mehmood et al. [22]—Auxetic polymeric bone plate as internal fixator for long bone fractures: Design, fabrication and structural analysis	Fixation for long bones	Development of an auxetic bone plate to reduce stress shielding and increase micromotions
10	Kapnisi et al. [30]—Auxetic cardiac patches with tuneable mechanical and conductive properties toward treating myocardial infarction	Cardiac patches	Developed auxetic conductive cardiac patches for the treatment of myocardial infarction (MI)
11	Arjunan et al. [32]—3D printed auxetic nasopharyngeal swabs for COVID-19 sample collection	Nasopharyngeal swabs	Design of nasopharyngeal swabs to reduce the stress on the surrounding tissues in the nasal cavity during swab collection
12	Panico et al. [33]—Development of a biomedical neck brace through tailored auxetic shapes	Orthoses, bandages, orthopaedic insoles	Development of a neck brace for orthopaedic purposes
13	Hinrichs et al. [35]—Active auxetic heel support for Achilles tendon therapy, Senior Design Project Report	Orthoses, bandages, orthopaedic insoles	Development of flexible mesh materials with the ability to adjust their flexibility and strength to simulate and support muscles and tendons
14	Pattinson et al. [36]—Additive manufacturing of biomechanically tailored meshes for compliant wearable and implantable devices	Orthoses, bandages, orthopaedic insoles	Presenting approach to digital fabrication of biomechanically tailored auxetic mesh materials (ankle brace) using AM
15	Mottram et al. [37]—US Patent for cranial remoulding orthosis and method of manufacture thereof (Patent # 10,695,211)	Orthoses, bandages, orthopaedic insoles	The purpose of the invention is to eliminate some of the disadvantages of existing orthoses, as well as to create an economical, effective, and hygienic orthosis for correcting head deformities
16	Moroney et al. [38]—Application of auxetic material for protective sports apparel	Sport protection	Investigate auxetic materials potential for enhanced wearer functionality through properties of synclastic curvature and biaxial expansion
17	Chen et al. [39]—Cyclic tensile stimulation enrichment of Schwann cell-laden auxetic hydrogel scaffolds towards peripheral nerve tissue engineering	Scaffolds	Development auxetic hydrogel scaffolds and studied cyclic tensile stimulation effects on the neural differentiation capabilities of human Schwann cells
18	Flamourakis et al. [40]—Laser-made 3D Auxetic Metamaterial Scaffolds for Tissue Engineering Applications	Scaffolds	Development adaptable auxetic scaffolds for tissue engineering applications
19	Yan et al. [41]—Pluripotent stem cell expansion and neural differentiation in 3-D scaffolds of tuneable Poisson’s ratio	Scaffolds	Evaluate the ability of the 3-D auxetic scaffolds with tuneable biophysical properties (*E* and ν) to influence PSC expansion and neural differentiation
20	Song et al. [42]—Vascular differentiation from pluripotent stem cells in 3-D auxetic scaffolds	Scaffolds	Demonstrated 3D auxetic scaffolds for vascular differentiation and provides a platform to study the influence of biophysical microenvironments on differentiation of pluripotent stem cells
21	Díaz Lantada et al. [43]—Auxetic tissue engineering scaffolds with nanometric features and resonances in the megahertz range	Scaffolds	Presenting an approach for the development of auxetics based on the use of deep reactive ion etching (DRIE)
22	Soman et al. [45]—Spatial tuning of negative and positive Poisson’s ratio in a multi-layer scaffold	Scaffolds	Developing of hybrid scaffolds for integration with human mesenchymal stem cells
23	Pagliara et al. [46]—Auxetic nuclei in embryonic stem cells exiting pluripotency Nature Materials	Auxetic structures and membranes for in vitro medical devices	Study of the mechanical phenotype of the metastable phenotype of the transition of ESCs the study of the nuclear response to compressive and tensile forces
24	Warner et al. [48]—3D-printed biomaterials with regional auxetic properties	Scaffolds	Developing scaffolds to aid in tendon-to-muscle tissue regeneration, i.e., appropriate scale for clinical tissue replacement, unit-cell architectures capable of supporting aggregate cell growth, and tuneable auxetic kinematics with actuation and mechanical energy storage capabilities that mimic tendon behaviour
25	Xue et al. [49]—Design of self-expanding auxetic stents using topological optimization	Stents/Simulation and topology optimization	Develop a topology optimization method
26	Auricchio et al. [50]—A novel layered topology of auxetic materials based on the tetrachiral honeycomb microstructure	Simulation and topology optimization	Design, test and compare bi-tetrachiral structure with tetrachiral structure, using both solid and beam lattice models
27	Wilt et al. [51]—Accelerating auxetic metamaterial design with Deep Learning	Simulation and topology optimization	Develop an auxetic material design process based on predictions by FEA and deep learning
28	Nguyen et al. [52]—Conformal lattice structure design and fabrication	Computational prediction and AI-aided design	Develop a method of CAD-generation of MSLS for complex-shaped parts
29	Engelbrecht et al. [53]—Design of meso-scale cellular structure for rapid manufacturing	Computational prediction and AI-aided design	Develop a method to generate one or more layers of meso-scale cellular structure for a given surface
30	Chu et al. [54]—A comparison of synthesis methods for cellular structures with application to additive manufacturing	Computational prediction and AI-aided design of new auxetic	Comparison of two synthesis methods, Particle Swarm Optimization (PSO) and least-squares minimization (LSM), for the design of components comprised of cellular structures
31	Dagdelen et al. [57]—Computational prediction of new auxetic materials	Simulation and topology optimization	Develop a database screening process to find non-organic crystalline materials with auxetic properties
32	Vinay et al. [58]—Fabrication and Testing of Auxetic Foams for Rehabilitation Applications	Traditional methods of manufacturing auxetics	Exploring auxetic foams for suitability in various rehabilitation applications
33	Plant et al. [59]—Injection mouldable rate stiffening re-entrant cell arrays for wearable impact protection	Traditional methods of manufacturing auxetics/Sport protection	Describing design and testing of affordable personal protection using an intrinsically impact-mitigating auxetic structure, moulded from rate stiffening thermoplastic blend
34	Ghaedizadeh et al. [60]—Tuning the performance of metallic auxetic metamaterials by using buckling and plasticity	Traditional methods of manufacturing auxetics	Guideline for the design of 2D metallic auxetics for various applications
35	Taylor et al. [61]—Low Porosity Metallic Periodic Structures with Negative Poisson’s Ratio	Traditional methods of manufacturing auxetics	Investigate the effect of the hole aspect ratio on the macroscopic Poisson’s ratio
36	Grujicic et al. [62]—multi-physics modelling of the fabrication and dynamic performance of all-metal auxetic-hexagonal sandwich-structures	Rolling, casting, and foaming methods of manufacturing auxetics	Investigate the effect of the prior processing and the resulting microstructure on the performance of all-metal sandwich-structures with an auxetic-hexagonal core
38	Ali et al. [29]—Auxetic polyurethane stents and stent-grafts for the palliative treatment of squamous cell carcinomas of the proximal and mid oesophagus: a novel fabrication route	Stents, rolling, casting, 3D printing and foaming methods of manufacturing auxetics	Development of a small diameter auxetic stent to the palliative treatment of squamous cell carcinomas of the oesophagus and for the prevention of dysphagia
39	Scarpa et al. [63]—Auxetic compliant flexible PU foams: static and dynamic properties	Rolling, casting, and foaming methods of manufacturing auxetics	Manufacturing of auxetic thermoplastic polyurethane foams
40	Jiang et al. [64]—A study of tubular braided structure with negative Poisson’s ratio behaviour	Textile based processes manufacturing of auxetics	Development of auxetic production process by modifying the pipe braiding technology.
41	Sloan et al. [65]—The helical auxetic yarn—A novel structure for composites and textiles; geometry, manufacture, and mechanical properties	Textile based processes manufacturing of auxetics	Describing the manufacture of monofilament HAYs and mechanical characterization process in detail and identify the mechanism behind the observed auxetic behaviour
42	Bhattacharya et al. [66]—The variation in Poisson’s ratio caused by interactions between core and wrap in helical composite auxetic yarns	Textile based processes manufacturing of auxetics	Study of the helical auxetic yarn via characterization of a wide range of polymeric fibres and yarns
43	Zhou et al. [67]—Auxetic composites made of 3D textile structure and polyurethane foam	Textile based processes manufacturing of auxetics	Development 3D auxetic textile composites for different potential applications
44	Vyavahare et al. [75]—Re-entrant auxetic structures fabricated by fused deposition modelling: An experimental study of influence of process parameters under compressive loading	3D-printing methods (FDM, SLM, SLS, stereolithographic methods) manufacturing of auxetics	Investigating the influence of process parameters under compressive loading of auxetic structures
45	Alomarah et al. [76]—An investigation of in-plane tensile properties of re-entrant chiral auxetic structure	3D-printing methods (FDM, SLM, SLS, stereolithographic methods) manufacturing of auxetics	Investigation of mechanical properties a new auxetic structure, named RCA
46	Geng et al. [77]—Mechanical properties of Selective Laser Sintering (SLS) additive manufactured chiral auxetic cylindrical stent	3D-printing methods (FDM, SLM, SLS, stereolithographic methods) manufacturing of auxetics/Stents	Fabrication of hybrid chiral stent with auxetic properties by additive manufacturing technique. Including investigation In-plane theoretical and experimental mechanical properties of stents
47	Kolken et al. [78]—Mechanical performance of auxetic meta-biomaterial	3D-printing methods (FDM, SLM, SLS, stereolithographic methods) manufacturing of auxetics	Investigation mechanical properties of auxetic meta-biomaterials
48	Xue et al. [79]—Compressive property of Al-based auxetic lattice structures fabricated by 3-D printing combined with investment casting	3D-printing methods (FDM, SLM, SLS, stereolithographic methods) manufacturing of auxetics	Investigation relationship between the structure and properties of auxetic structure
49	Cheng et al. [82]—3D printing-directed auxetic Kevlar aerogel architectures with multiple functionalization options	3D-printing methods (FDM, SLM, SLS, stereolithographic methods) manufacturing of auxetics	Designing and fabrication of aerogel auxetic architectures
50	Tang et al. [84]—Highly tailorable electromechanical properties of auxetic piezoelectric ceramics with ultra-low porosity	3D-printing methods (FDM, SLM, SLS, stereolithographic methods) manufacturing of auxetics/Simulation and topology optimization	Investigation of electromechanical properties of auxetic piezoelectric ceramic with ultra-low porosity
